# Evaluating the efficacy and safety of nivolumab and ipilimumab combination therapy compared to nivolumab monotherapy in advanced cancers (excluding melanoma): a systemic review and meta-analysis

**DOI:** 10.1186/s43046-024-00218-2

**Published:** 2024-05-06

**Authors:** Hussain Sohail Rangwala, Hareer Fatima, Mirha Ali, Sailesh Sunder, Sonia Devi, Burhanuddin Sohail Rangwala, Syed Raza Abbas

**Affiliations:** 1 Department of Medicine, Jinnah Sindh Medical University, Karachi, Pakistan; 2 Department of Medicine, Shaheed Mohtarma Benazir Bhutto Medical College Lyari, Karachi, Pakistan; 3 Department of Medicine, Ghulam Muhammad Mahar Medical College, Karachi, Pakistan; 4 Department of Medicine, Dow University of Health Sciences, Karachi, Pakistan

**Keywords:** Nivolumab, Ipilimumab, Combination therapy, Overall survival, Progression-free survival, Adverse events

## Abstract

**Background:**

Nivolumab (Nivo) and ipilimumab (Ipi) have revolutionized cancer treatment by targeting different pathways. Their combination shows promising results in various cancers, including melanoma, but not all studies have demonstrated significant benefits. A meta-analysis was performed to assess the effectiveness and safety of Nivo-Ipi compared to Nivo alone in advanced cancer types (excluding melanoma).

**Methods:**

Following PRISMA guidelines, we conducted a meta-analysis up to September 30, 2023, searching databases for randomized controlled trials (RCTs). We focused on advanced solid malignancies (excluding melanoma) with specific Nivo and Ipi dosing. Primary outcomes were overall survival (OS), progression-free survival (PFS), grades 3–4 adverse events (AEs), and treatment-related discontinuations. Secondary outcomes included specific adverse events. Statistical analysis in Review Manager included hazard ratio (HR) and risk ratio (RR), assessing heterogeneity (Higgins *I*^2^).

**Results:**

Nine RCTs, involving 2152 patients covering various malignancies, were analyzed. The Nivo plus Ipi group exhibited a median OS of 12.3 months and a median PFS of 3.73 months, compared to monotherapy with 11.67 months and 3.98 months, respectively. OS showed no significant difference between Nivo and Ipi combination and Nivo alone (*HR* = 0.97, 95% *CI*: 0.88 to 1.08, *p* = 0.61). PFS had a slight improvement with combination therapy (*HR* = 0.91, 95% *CI*: 0.82 to 1.00, *p* = 0.04). Treatment-related cumulative grades 3–4 adverse events were higher with Nivo and Ipi (*RR* = 1.52, 95% *CI*: 1.30 to 1.78, *p* < 0.00001), as were treatment-related discontinuations (*RR* = 1.99, 95% *CI*: 1.46 to 2.70, *p* < 0.0001). Hepatotoxicity (*RR* = 2.42, 95% *CI*: 1.39 to 4.24, *p* = 0.002), GI toxicity (*RR* = 2.84, 95% *CI*: 1.44 to 5.59, *p* = 0.002), pneumonitis (*RR* = 2.29, 95% *CI*: 1.24 to 2.23, *p* = 0.008), dermatitis (*RR* = 2.96, 95% *CI*: 1.08 to 8.14, *p* = 0.04), and endocrine dysfunction (*RR* = 6.22, 95% *CI*: 2.31 to 16.71, *p* = 0.0003) were more frequent with Nivo and Ipi.

**Conclusions:**

Combining nivolumab and ipilimumab did not significantly improve overall survival compared to nivolumab alone in advanced cancers (except melanoma). However, it did show slightly better PFS at the cost of increased toxicity, particularly grades 3–4 adverse events. Specific AEs occurred more frequently in the combination group. Further trials are needed to fully assess this combination in treating advanced cancers.

## Background

Immune checkpoint inhibitors, nivolumab (Nivo) and ipilimumab (Ipi), have revolutionized cancer treatment. Nivo targets programmed death-ligand 1 (PD-L1), while Ipi inhibits anti-cytotoxic T-lymphocyte antigen 4, and they complement each other in their mechanisms of action [1]. Notably, combining Nivo and Ipi has demonstrated impressive advancements in both progression-free survival (PFS) and overall survival (OS), particularly in metastatic melanoma. This is particularly striking in cases of v-raf murine sarcoma viral oncogene homolog B1mutation-positive and PD-L1-negative melanomas, when compared to Nivo monotherapy [2, 3]. The efficacy of the Nivo-Ipi combination extends beyond melanoma, with long-term overall survival benefits observed in various types of cancers like esophageal squamous cell carcinoma, malignant pleural mesothelioma, renal cell carcinoma, and non-small cell lung cancer (NSCLC) [3–7]. Although there is a dearth of published trials directly comparing the Nivo-Ipi combination to Nivo alone for advanced malignancies aside from melanoma, the combination is generally considered as a superior treatment option for malignancies. Several trials have explored its potential to outperform the current standard of care across different cancer types [8]. Nevertheless, it is crucial to acknowledge that a few trials, such as the phase 3 CheckMate 651 trial [9], have not shown significant clinical benefits in terms of overall survival with this combination therapy. To address this discrepancy, we aim to perform a comprehensive meta-analysis of available literature comparing the effectiveness and safety of the Nivo-Ipi combination compared to Nivo alone in advanced cancer types, excluding melanoma.

## Methods

### Data sources and search strategy

This meta-analysis adhered to the PRISMA guidelines [10]. To ensure a thorough investigation for our analysis, we conducted searches in two databases, PubMed and the Cochrane Library, covering studies published up to September 30, 2023. Employing these two prominent databases was our strategy to reduce the potential impact of publication bias. Our search approach involved meticulously constructing a search string to identify studies relevant to our research. The search string included various combinations of key terms such as “nivolumab,” “ipilimumab,” and “nivolumab and ipilimumab,” and articles were retrieved and identified manually for further evaluation.

### Inclusion criteria

The study’s inclusion criteria were meticulously established and guided by specific parameters. Firstly, we focused on only randomized controlled trials (RCTs) with publication dates up to September 30, 2023. Secondly, our selection criteria required that the chosen studies encompass patients diagnosed with metastatic or advanced solid malignancies (except melanoma). Within the realm of eligible studies, investigations involving the following specific dose regimens for intervention were considered: Nivo at a dose of 3 mg per kilogram every 2 weeks, combined with ipilimumab at 1 mg per kilogram every 6 weeks until disease advancement. Additionally, studies adopting an alternate regimen in which Nivo is administered at a rate of 3 mg per kilogram every 3 weeks, in conjunction with ipilimumab at 1 mg per kilogram every 3 weeks, for a total of four doses, were also considered. For the control group, we encompassed those administering nivolumab at a rate of 3 mg per kilogram every 2 weeks and those utilizing a flat-dosing regimen of Nivo at 240 mg every 2 weeks.

The exclusion criteria were as follows: (1) studies solely focused on metastatic or advanced melanoma, (2) studies or specific arms within studies that employed combination dosing of Nivo and Ipi at doses other than Nivo at 3 mg per kilogram and Ipi at 1 mg per kilogram regimen, as described above, (3) the dosing regimen of Nivo (1 mg per kilogram) plus Ipi (3 mg per kilogram) is recognized for its higher toxicity compared to Nivo at a dose of 3 mg per kilogram combined with Ipi at 1 mg per kilogram. It has received approval for use in just one type of cancer, hepatocellular carcinoma, and as a result, it was omitted from the analysis to maintain consistency in the study.

### Outcome measures

The primary outcome measures were overall survival (OS), progression-free survival (PFS), grades 3 or 4 adverse events (AEs), and treatment-related discontinuations. Secondary outcomes were grades 3–4 adverse hepatotoxicity events, gastrointestinal toxicity, pneumonitis, endocrine dysfunction, and dermatitis.

### Data extraction and quality assessment

In the initial phase of our study selection process, we screened titles and abstracts to exclude any studies that did not align with our predefined eligibility criteria. We effectively employed the EndNote Reference Library program to manage and prevent duplication of articles. Subsequently, we retrieved full-text articles for a comprehensive evaluation to determine their suitability for inclusion in our meta-analysis.

To maintain rigor in the data extraction process, three authors collectively participated in extracting pertinent data from each of the selected RCTs. The extracted data encompassed critical details such as the primary author’s name, year of publication, research methodology, patient population attributes, trial phase, study title, administered treatments, patient distribution among treatment arms, total patient count in the study, median patient age within each intervention group, median OS, PFS along with their respective 95% confidence intervals (CIs), treatment-related discontinuations, and a comprehensive account of grades 3–4 AEs.

To gauge the included study’s overall quality in our analysis, two authors diligently utilized the Cochrane risk-of-bias tool for randomized trials (RoB 2) [11]. This tool was instrumental in assessing the potential bias risk inherent in the included studies. In cases where differences in assessments arose, they were resolved through consensus or, if necessary, by consulting a third investigator to ensure a rigorous and unbiased evaluation of the studies.

### Statistical analysis

For statistical analysis, we utilized the Review Manager software package version 5.4.1. Our primary objective was to ascertain the significance of the combination therapy involving Nivo and Ipi compared to Nivo monotherapy. A systematic review was conducted to accomplish this, presenting the findings qualitatively and quantitatively through a meta-analysis of pooled hazard ratios (HR) and risk ratios (RR) with 95% confidence intervals (95% Cls). To assess the statistical heterogeneity across the included studies, Higgins *I*^2^ statistic was employed within a random-effects model. The random-effects model makes a less stringent assumption compared to the fixed-effects model. Instead of assuming a single true effect size that is common to all studies, the random-effects model allows for variability in study effect sizes. In this perspective, there is not a singular effect size; rather, multiple effect sizes are acknowledged. The underlying assumption is that the distribution of these study effect sizes follows a pattern centered around the true effect size of interest [12]. An *I*^2^ value of 25% or greater signifies low heterogeneity, whereas values falling between 50 and 75% indicate moderate heterogeneity, and values surpassing 75% suggest high heterogeneity. This comprehensive approach ensured the precision and reliability of our statistical analysis in evaluating the therapeutic effects of Nivo and Ipi in comparison to Nivo alone.

## Results

### Studies selection

**Fig. 1:**
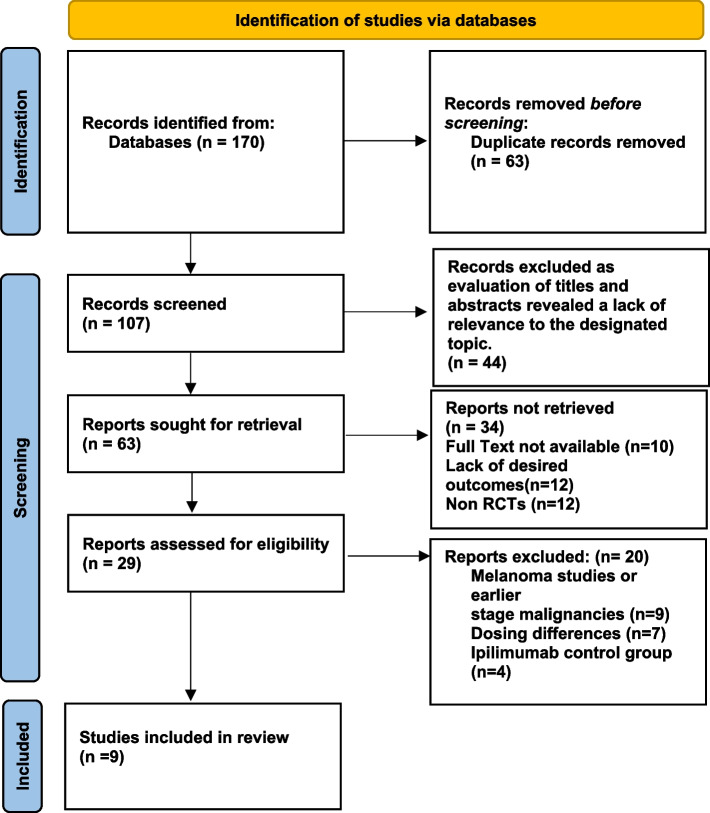
PRISMA flow diagram

Our initial search yielded 170 studies. Subsequently, 63 duplicate records were identified and subsequently eliminated. Among the remaining 107 studies, 44 were excluded based on irrelevance. The remaining 63 studies were chosen for further evaluation due to their relevance to the subject matter. Following a thorough assessment, 34 additional studies were excluded—consequently, our final selection for inclusion in the meta-analysis comprised of 9 RCTs [13–21] (Fig. 1).

### Study characteristics

Nine studies, comprising a total of 2152 patients, were eligible based on the selection criteria. Among these, 1134 patients were in the Nivo plus Ipi group, while 1016 were in the Nivo monotherapy group. The patients in these studies had various types of malignancies, including small cell lung cancer [13], sarcoma [14], glioblastoma multiforme [15], esophagogastric carcinoma [16], pleural mesothelioma [17], urothelial carcinoma [18], NSCLC [19], squamous cell lung cancer [20], and squamous cell carcinoma of the head and neck [21]. The median OS for the Nivo plus Ipi group was 12.3 (6.55) months, while for monotherapy, it was 11.67 (7.03) months. In terms of PFS, the combination group had a median of 3.73 (2.18) months, and the monotherapy group had a median PFS of 3.98 (5.7) months (Table 1).

**Table 1:** Attributes of the shortlisted studies

Study name and year	Phase	Randomized	Open label	Single center/multicenter	Disease	Patient population in (I/C)^a^	Intervention	Control	Median age, year		Median OS, months (95% *CI*)		Median PFS, months (95% *CI*)	
									***I***	***C***	***I***	***C***	***I***	***C***
Harrington et al. (2023) [21]	2	Yes	No	Multicenter	R/M squamous cell carcinoma of the head and neck	425 (280/143)	A	C	59.9	68.8	10.3 (6–14.5)	9 (6.7–13.3)	2.7 (1.4–2.8)	2.6 (1.4–4.1)
Gettinger et al. (2021) [20]	3	Yes	Yes	Multicenter	M squamous cell lung cancer	252 (125/127)	A	C	67.5	68.1	NR^b^	NR^b^	28.4 (4.9–NE)	9.7 (4.2–23.1)
Hellmann et al. (2019) [19]	3	Yes	Yes	Multicenter	M/R NSCLC with PD-L1 ≥ 1%	792 (396/396)	A	D	64	64	17.1 (15.2–19.9)	15.7 (13.3–18.1)	5.1 (4.1–6.3)	4.2 (3–5.3)
Scherpereel et al. (2019) [17]	2	Yes	Yes	Multicenter	Relapsed pleural mesothelioma	125 (62/63)	A	C	71.2	72.3	15.9 (10.7–N/A)	11.9 (6.7–17.7)	5.6 (3.1–8.3)	4 (2.8–5.7)
Sharma et al. (2019) [18]	1/2	No	Yes	Multicenter	M urothelial carcinoma	182 (104/78)	B	C	63	65.5	7.4 (5.6–11)	9.9 (7.3–21.1)	2.6 (1.4–3.9)	2.8 (1.8–5.3)
Janjigian et al. (2018) [16]	1/2	No	Yes	Multicenter	M esophagogastric carcinoma	111 (52/59)	B	C	58	60	4.8 (3.8–8.4)	6.2 (3.4–12.4)	1.6 (1.4–2.6)	1.4 (1.2–1.5)
D’Angelo et al. (2018) [14]	2	Yes	Yes	Multicenter	Advanced sarcoma	83 (41/42)	B	C	57	56	14.3(9.6–NE)	10.7 (5.5–15.4)	4.1 (2.6–4.7)	1.7 (1.4–4.3)
Omuro et al. (2018) [15]	1	No	Yes	Multicenter	Glioblastoma multiforme	30 (20/10)	B	C	60	58.5	7.3 (4.7–12.9)	10.4 (4.11–22.8)	2.1 (1.4–2.8)	1.9 (1.3–4.6)
Antonia et al. (2016) [13]	1/2	No	Yes	Multicenter	R small cell lung cancer	152 (54/98)	B	C	61	63	6 (3.6–11)	4.4 (3–9.3)	1.4 (1.3–2.2)	1.4 (1.4–1.9)

*Abbreviations*: *M *Metastatic, *R *Recurrent, *NSCLC *Non-small cell lung cancer, *PD-L1 *Programmed death ligand 1, *I *Intervention, *C *Control, *OS *Overall survival, *PFS *Progression-free survival, *NR *Not reported, *NE *Not estimable

^a^The overall number of patients includes those receiving ipilimumab and standard-dose nivolumab but not those receiving alternative therapies

A, nivolumab at a dose of 3 mg per kilogram every 2 weeks, combined with ipilimumab at 1 mg per kilogram every 6 weeks until disease advancement

B, nivolumab is administered at a rate of 3 mg per kilogram every 3 weeks, in conjunction with ipilimumab at 1 mg per kilogram every 3 weeks, for a total of four doses. This is followed by a maintenance phase of nivolumab at 3 mg per kilogram every 2 weeks

C, administering nivolumab at a rate of 3 mg per kilogram every 2 weeks

D, 240 mg of nivolumab given every 2 weeks. *Only hazard ratio was reported

^b^Only hazard ratio was reported

### Quality assessment

**Fig. 2:**
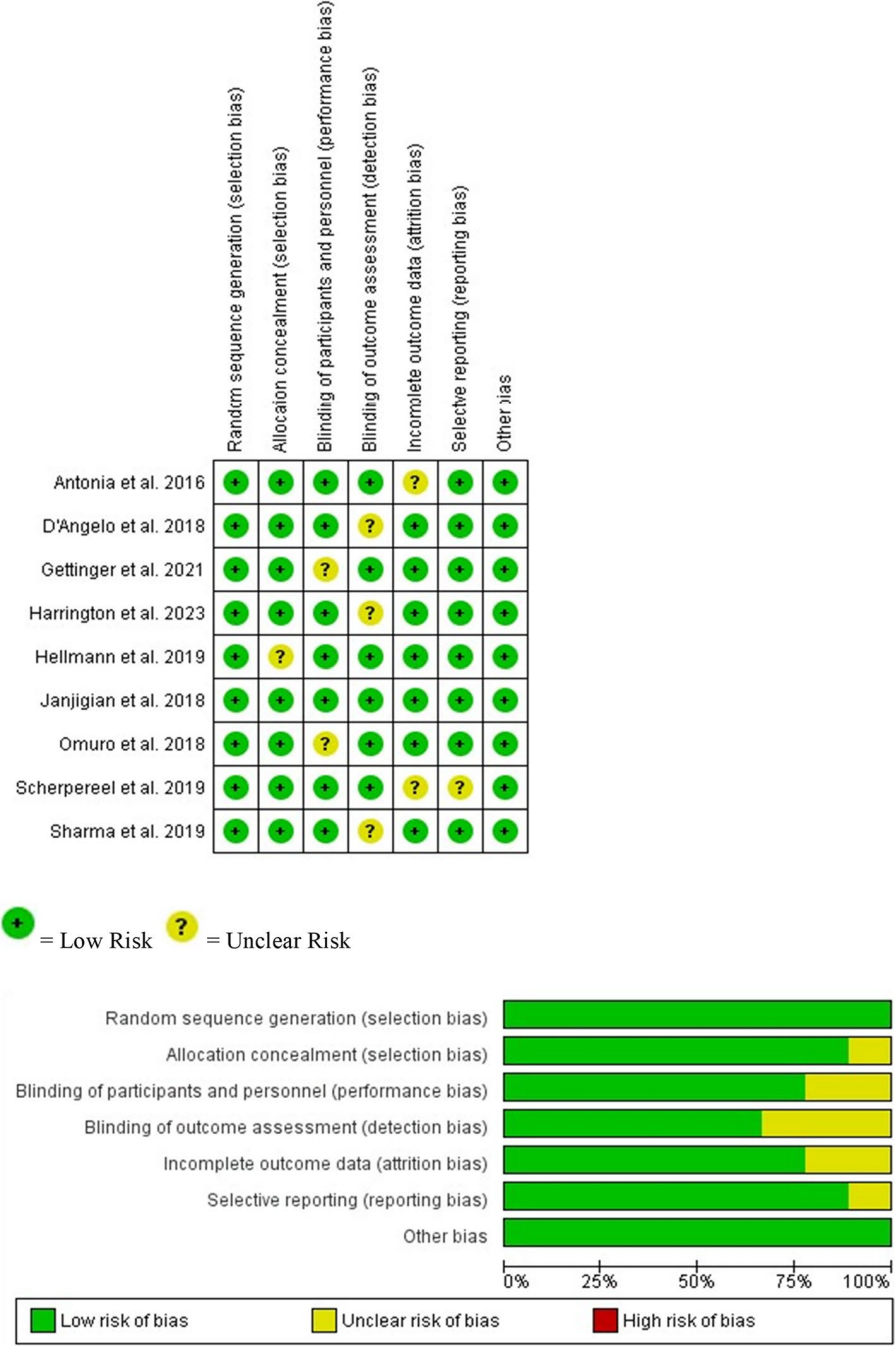
Quality judgments about each risk-of-bias item

We employed the RoB 2 [11] to assess the included studies, and the results are illustrated in Fig. 2. Notably, all of the studies in our analysis were determined to exhibit minimal risk of bias, underscoring their high level of reliability.

### Overall survival

**Fig. 3:**
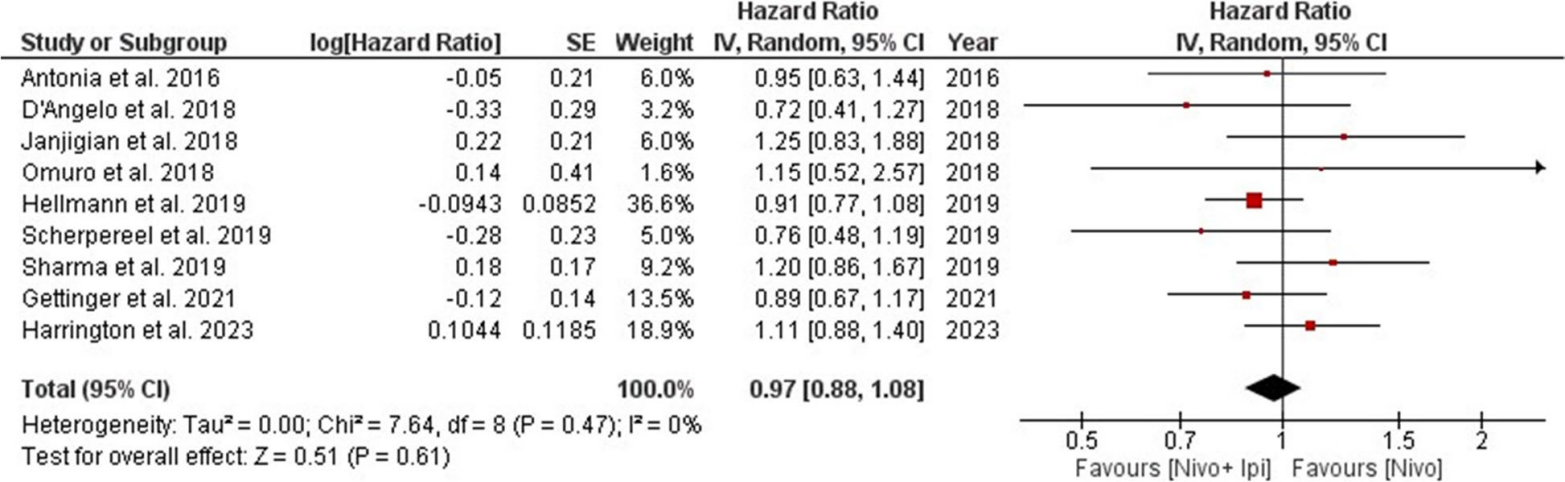
Forest plot for the comparison of OS

The combined hazard ratio (HR) derived from the analysis of nine studies was calculated using the random-generic inverse variance method. It revealed an almost similar risk of overall survival (OS) between the combination of Nivo and Ipi and Nivo alone (*HR* = 0.97; 95% confidence interval: 0.88 to 1.08, *p* = 0.61) (*I*^2^ = 0%, *p* = 0.47). These findings were based on data from 2152 patients across the 9 studies, indicating no significant difference between the 2 medications (Fig. 3).

### Progression-free survival

**Fig. 4:**
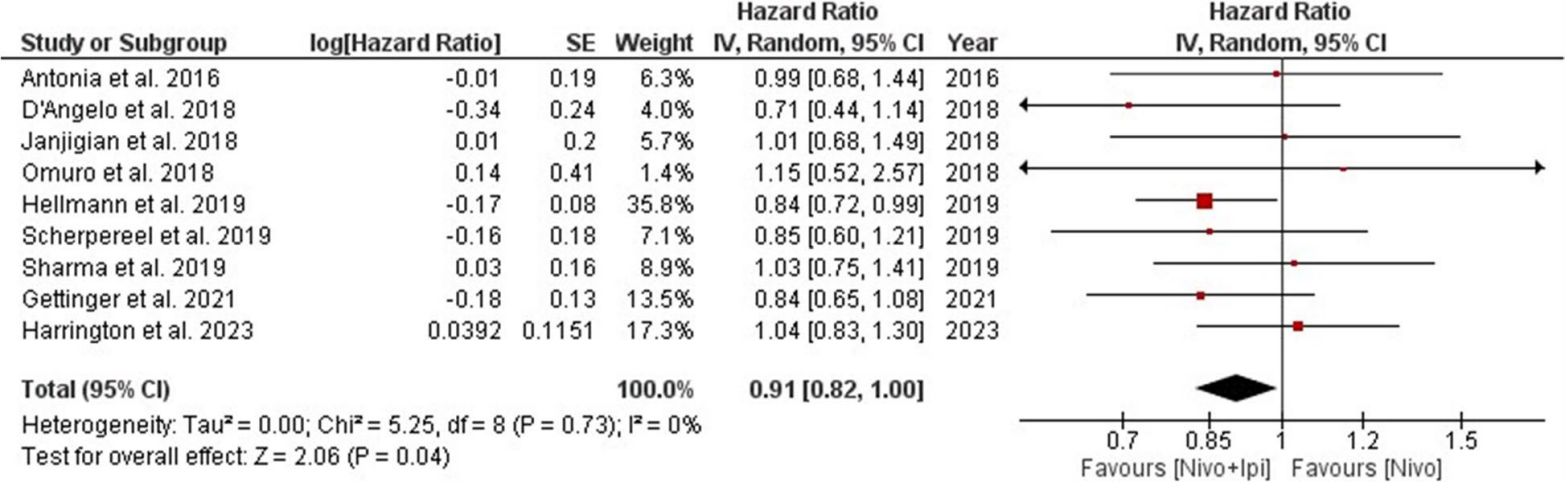
Forest plot for the comparison of PFS

The combined hazard ratio (HR) calculated using the random-effects generic inverse variance method showed a slight decrease in risk when comparing the combination of Nivo and Ipi to Nivo alone in terms of progression-free survival (PFS) (*HR* = 0.91; 95% confidence interval: 0.82 to 1.00, *p* = 0.04) (*I*^2^ = 0%, *p* = 0.73). These results were drawn from data analysis from 9 studies encompassing 2152 patients (Fig. 4).

### Treatments related to cumulative grades 3–4 adverse events

**Fig. 5:**
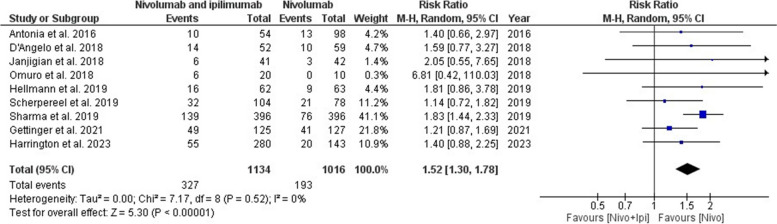
Forest plot for the comparison of treatments related to cumulative grades 3–4 adverse events

Using the random-effects inverse variance method, we computed a combined relative risk (RR) for the incidence of cumulative grades 3–4 adverse events associated with treatments, drawing data from nine distinct studies. The findings revealed a significant increase in treatment-related cumulative grades 3*–*4 adverse events when comparing the combination of Nivo and Ipi to Nivo alone, with an observed RR of 1.52 (95% confidence interval: 1.30 to 1.78, *p* < 0.00001) (*I*^2^ = 0%, *p* = 0.52) (Fig. 5).

### Treatment-related discontinuations

**Fig. 6:**
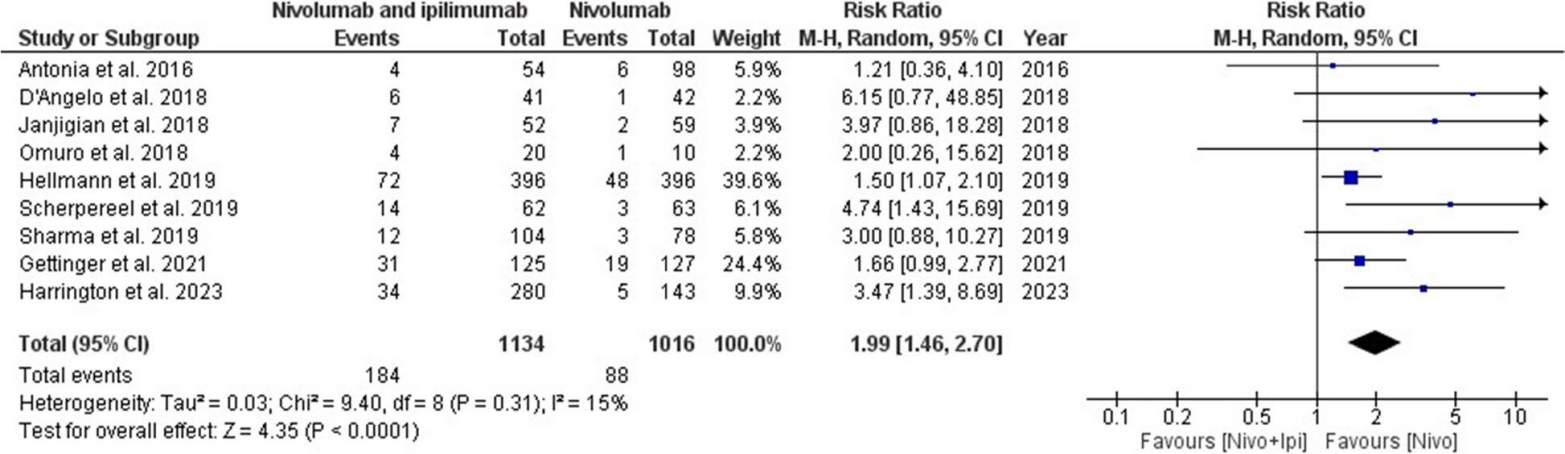
Forest plot for the comparison of treatment-related discontinuations

The analysis indicates a substantial increase in the incidence of treatment-related discontinuations when the combination of Nivo and Ipi is used compared to using Nivo as a monotherapy. The observed relative risk (RR) is 1.99 (95% *CI*: 1.46 to 2.70, *p* < 0.0001) (*I*^2^ = 15%, *p* = 0.31) (Fig. 6).

### Grades 3–4 hepatotoxicity

**Fig. 7:**
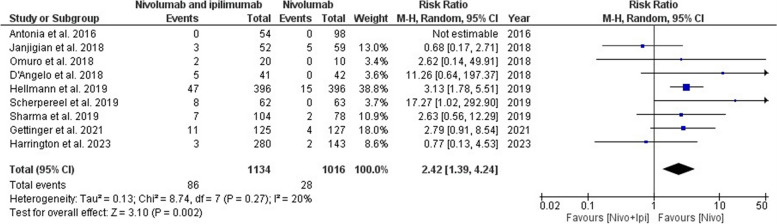
Forest plot for the comparison of grades 3–4 hepatotoxicity

Nivo and Ipi combination was associated with a significantly elevated incidences of hepatoxicity when compared to Nivo alone, with an observed relative risk (RR) of 2.42 (95% *CI*: 1.39 to 4.24, *p* = 0.002) (*I*^2^ = 20%, *p* = 0.27) (Fig. 7).

### Grades 3–4 GI toxicity

**Fig. 8:**
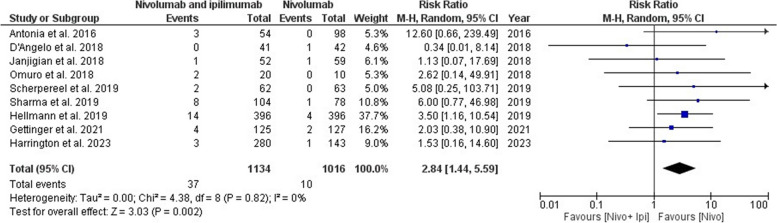
Forest plot for the comparison of grades 3–4 gastrointestinal (GI) toxicity

The combination of Nivo and Ipi demonstrated a significantly higher incidence of gastrointestinal (GI) toxicity compared to Nivo alone, with an observed relative risk (RR) of 2.84 (95% *CI*: 1.44 to 5.59, *p* = 0.002) (*I*^2^ = 0%, *p* = 0.82) (Fig. 8).

### Grades 3–4 pneumonitis

**Fig. 9:**
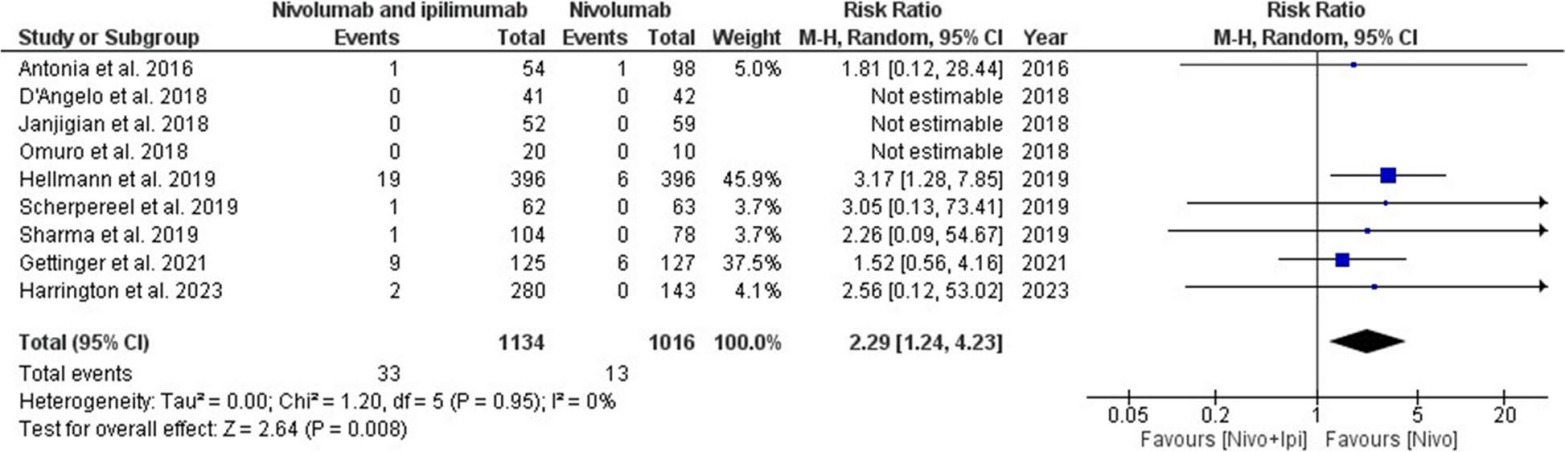
Forest plot for the comparison of grades 3–4 pneumonitis. Nivo, nivolumab; Ipi, ipilimumab

The combination of Nivo and Ipi exhibited a higher occurrence of pneumonitis in comparison to Nivo alone, with an observed relative risk (RR) of 2.29 (95% *CI*: 1.24 to 2.23, *p* = 0.008) (*I*^2^ = 0%, *p* = 0.95) (Fig. 9).

### Grades 3–4 dermatitis

**Fig. 10:**
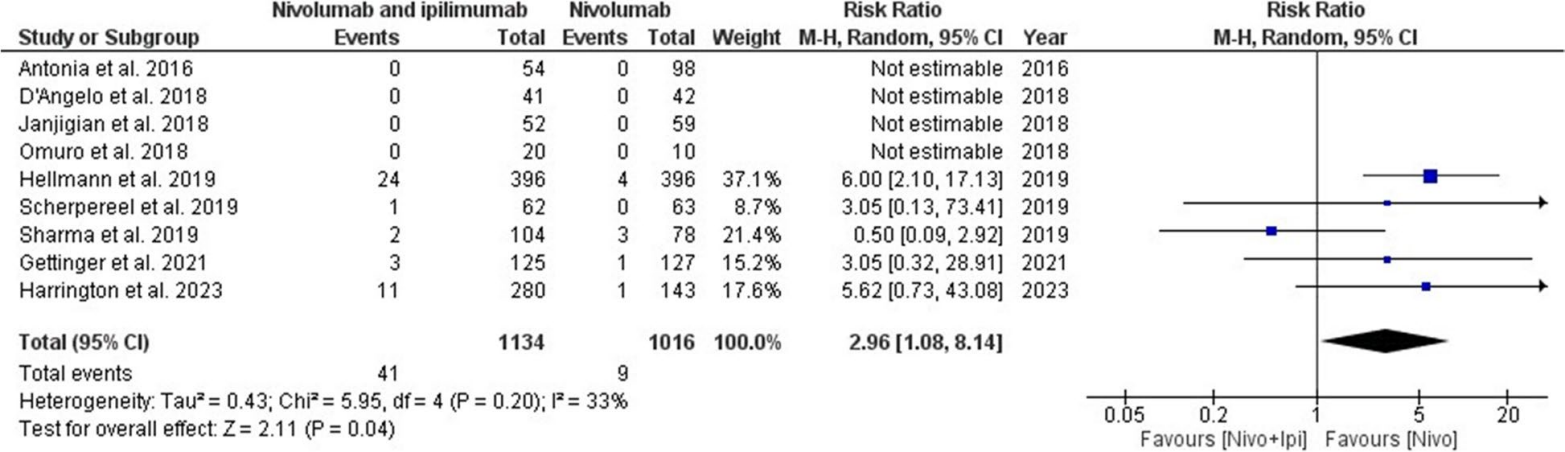
Forest plot for the comparison of grades 3–4 dermatitis

The combination therapy of Nivo and Ipi exhibited a higher frequency of dermatitis events in contrast to Nivo alone, with an observed relative risk (RR) of 2.96 (95% *CI*: 1.08 to 8.14, *p* = 0.04) (*I*^2^ = 33%, *p* = 0.20) (Fig. 10).

### Grades 3–4 endocrine dysfunction

**Fig. 11:**
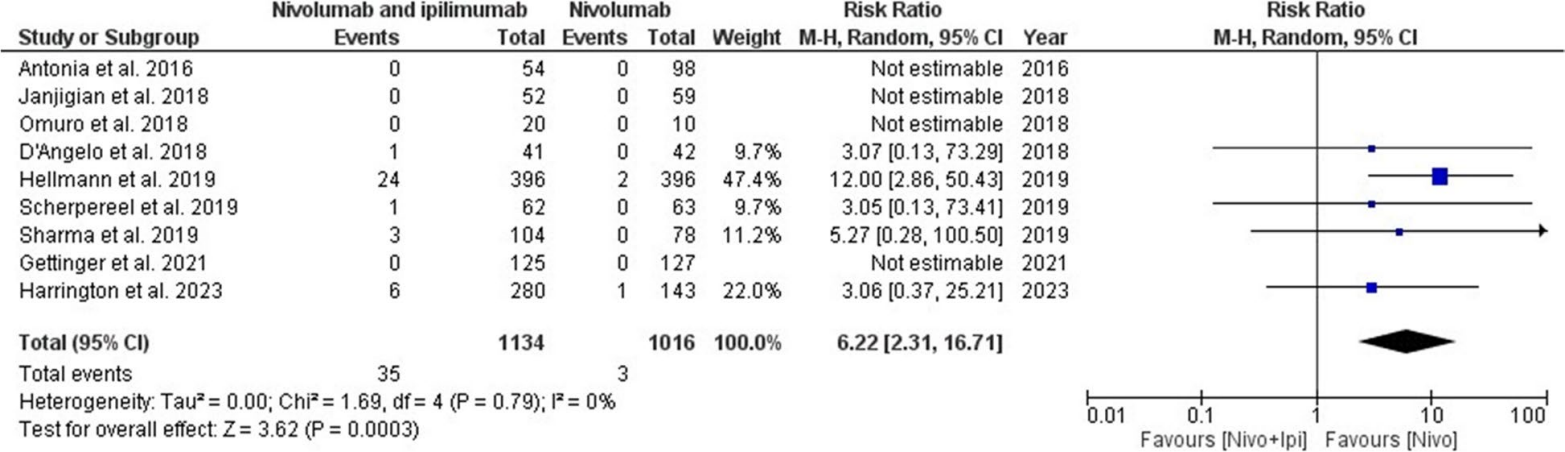
Forest plot for the comparison of grades 3–4 endocrine dysfunction

The combination of Nivo and Ipi resulted in a significantly elevated occurrence of endocrine dysfunction events when compared to Nivo alone, with an observed relative risk (RR) of 6.22 (95% *CI*: 2.31 to 16.71, *p* = 0.0003) (*I*^2^ = 0%, *p* = 0.79) (Fig. 11).

## Discussion

In the context of advanced malignancies other than melanoma, the tango that exists between Nivo-Ipi combination or Nivo monotherapy treatment is an ongoing debate that remains prominent in the healthcare field. With this in mind, each study helps to create a clearer picture by advancing our understanding of the best mode of treatment, weaving it together with the findings of previous studies. Our comprehensive meta-analysis aims to address the gap in literature regarding the efficacy and safety of combination therapy in providing significant clinical benefits in terms of overall survival, which has shown conflicting results in the past studies. This gap is filled in by adding a recent phase 2 CheckMate 714 trial [21], in addition to the previous eight studies [13–20]. With the inclusion of 425 more patients in the analysis pool, this study intends to evaluate the dual immunotherapy of Nivo-Ipi compared to Nivo monotherapy, thereby assessing the contribution of each component of dual immunotherapy as first-line treatment for patients with advanced cancer.

The combination of Nivo and Ipi, compared to Nivo alone, when evaluating overall survival rate, exhibits a nonsignificant relation between the two treatment groups, defying the previous study results that supported in favor of the Nivo-Ipi combination therapy in terms of enhanced survival [4–7]. Since overall survival is the desired outcome, the risk of death in the group receiving combination therapy was nearly indistinguishable from that of the monotherapy group, with combination therapy resulting in only a marginal 3% increase in the risk of death; however, this difference is quite negligible and clinically insignificant between the two treatment groups. This finding may be explained by the fact that nivolumab and ipilimumab are both immune checkpoint inhibitors. Ipilimumab targets CTLA-4, while nivolumab inhibits PD-1 [22, 23]. It is possible that the combined blocking effect might not provide an additional benefit in terms of overall survival rate. Additionally, as indicated in the Shi Zhou (2019) study, this dual therapy administration synergistically enhances the immune-related toxic effects (immune-related adverse events) by amplifying the blockade affect and reducing the survival chances [24]. Moreover, the low heterogeneity exhibited indicates a low variability between the studies, signifying consistent and less chances of skewing of the outcome results.

The study revealed a slight 9% reduction in the risk of progression-free survival (PFS), favoring the combination of Nivo and Ipi over Nivo alone in patients with advanced carcinoma. While this statistically significant result suggests a potential benefit in slowing disease progression, the small effect size and marginally significant *p*-value (*p* = 0.04) underscore the need for cautious interpretation. The low heterogeneity across the nine included studies (*I*^2^ = 0%) adds to the reliability of the findings. However, to gain a more comprehensive understanding of the therapeutic impact, further research is needed to thoroughly explore the implications and potential benefits of the observed reduction in PFS risk [4–7]. Drug resistance in malignancies, for instance, can be one of the factors that can be brought on by monotherapies, enhancing PFS and making it less reliable comparatively. A recent medical publication, titled *Combination Therapy Against Multidrug Resistance*, discussed the potential of combination therapy in overcoming multidrug resistance, providing a broad spectrum of efficacy, better potency than the medications used in monotherapy [25]. Henceforth, combining treatments could stop or delay the development of resistance due to its amplified effect, reducing PFS. Based on this, further investigative trials are required to provide a much clearer picture for the contrasted results between OS and PFS outcomes.

Although the Nivo-Ipi dual therapy has shown lesser progression in disease, it shows an increase in incidence of treatment-related cumulative grades 3–4 AEs and discontinuations associated with treatment, when compared to Nivo alone. Hepatotoxicity, diarrhea, elevated lipase, weariness, and rash were the most frequent AEs linked to combination immunotherapy [26]. This could hypothesize a directly proportional relationship between severity of adverse effects and discontinuation of the combination therapy, suggesting its lack of safety. However, the severity and extent of adverse responses may vary according to the dosage, frequency, and mode of administration technique. Nevertheless, specified symptomatic treatments should be provided to combat particular AEs.

The secondary outcomes of this study showcase a significant association between the Nivo-Ipi and Nivo monotherapy group in terms of grades 3–4 adverse hepatotoxicity events, gastrointestinal toxicity, pneumonitis, endocrine dysfunction, and dermatitis; this questions its efficacy at the cost of its safety. To combat the higher toxicity associated with the combination treatment strategy, a striking balance should be obtained by lowering the Ipi dosage when combined with the standard dose of Nivo, to reduce the elevated immune-blocking effect. One study (D'Angelo et al., 2018) also supported the hypothesis that this combination therapy could be safer if Ipi were administered at a lower dose [14, 26]. This emphasizes the need of attaining optimal dosages that achieve the appropriate balance to establish a robust treatment approach for advanced carcinoma patients. It further urges the need to conduct more randomized investigations to subcategorize and divide Ipi into specified, lower dose regimen and then combine it with the standard Nivo dose, to find the ideal quantity required for each type of advanced-stage carcinoma.

### Limitations

This study has certain limitations. Firstly, to explore the underlying mechanisms and establish a cause-and-effect relationship between the two treatment groups and the outcomes, larger interventional studies are required. It is worth noting that although our meta-analysis had a sufficient number of studies included in the analysis, further large-powered studies are required to reach more prominent findings. Secondly, this meta-analysis caters to different types of malignancies, creating variability and unknowingly favoring the combination strategy group. Additionally, studies with lower Nivo dose (1 mg/kg) and higher Ipi dose (3 mg/kg) were not included in the pool, suggesting a proposed discrepancy that may change the outcomes and enhance the grades 3–4 adverse events if included [27]. Our study also faced a significant limitation due to the restricted number of trials available for analysis (nine studies). This constraint hindered our ability to assess publication bias through methods such as a funnel plot analysis. Lastly, Ipi dosage for CheckMate 714 was 1 mg per kilogram IV every 6 weeks. Despite the fact that Ipi was well tolerated in CheckMate 714, patients with R/M SCCHN may not have received the best dosage or timing [21]. Therefore, future studies should consider these loopholes to enhance the quality of the outcome results.

## Conclusions

Our results indicate that the combined treatment of standard nivolumab and ipilimumab did not significantly differ from nivolumab alone in terms of overall survival for advanced cancers beyond melanoma. However, a significant difference was observed in PFS, with the Nivo-Ipi combination slightly outperforming nivolumab alone, but at the expense of higher toxicity rates [28]. Importantly, our analysis identified significantly higher grades 3–4 adverse events and treatment discontinuations in the combined immunotherapy group. Additionally, the study reported an increased occurrence of severe hepatotoxicity, gastrointestinal toxicity, pneumonitis, endocrine issues, and dermatitis in combination group. These observations underscore the necessity for more robust RCTs to delve deeper into the effects and potential factors influencing the outcomes of Nivo-Ipi combination strategy versus nivolumab monotherapy in treating advanced cancers.

## Data Availability

Data available within the article. The authors confirm that the data supporting the findings of this study are available within the article.

## Abbreviations

Nivo: Nivolumab
Ipi: Ipilimumab
OS: Overall survival
PFS: Progression-free survival
PD-L1: Programmed death-ligand 1
NSCLC: Non-small cell lung cancer
RCTs: Randomized controlled trials
AEs: Adverse events
CIs: Confidence intervals
RoB 2: Cochrane risk-of-bias tool for randomized trials
HR: Hazard ratios
RR: Risk ratios
